# Supplementary material for: Evaluating the sustainability and long-term outcomes of the Home Care Support Intervention Program (HoSIP) to reduce loneliness among community-dwelling older adults: A two-year follow-up study
Source: PLoS One. 2025 Oct 29;20(10):e0331987. doi: 10.1371/journal.pone.0331987 (PMC12571258; doi:10.1371/journal.pone.0331987)
Supplement: Attachment — Submitted filename: Response to Reviewer 2.docx [file pone.0331987.s002.docx]

***Response to Reviewer 2:***

***Introduction***

1- This manuscript aims to evaluate the long-term effects of the HoSIP intervention and serves as a follow-up to the authors’ previous publication. The purpose of the study is not well stated. Was the study aimed at assessing the sustainability of the intervention after it was discontinued after two years? Or is the intervention still ongoing?

Thank you for your valuable comment. We have revised the title “Evaluating *the Sustainability and Long-Term Outcomes of the Home Care Support Intervention Program (HoSIP) to Reduce Loneliness among Community-dwelling Older Adults: A two-year follow-up study*”. Please refer to page 1, Line 1; page 2, lines 36-38.

**Method**

2- While the manuscript is well-referenced, the Methods section lacks sufficient detail. In particular, the description of the intervention is unclear. As, mentioned, it is not specified whether the intervention is ongoing or if it was limited to the initial 12-week period. Clarification is needed on whether this study is assessing the sustainability of the intervention's effects two years after its conclusion, or if the intervention has continued in some form during that time. Since the stated aim of the study is to examine the sustainability of the intervention, a more explicit explanation of the timeline and implementation status is essential.

Thank you for your valuable comment. We have revised Methods section. Please refer page 6, lines 138-150.

2- Additionally, the data analysis section requires further elaboration. Although the Results section lists the statistical tests used, it does not clearly indicate what comparisons were made or what hypotheses were being tested. Providing this context would greatly enhance the interpretability and rigor of the findings.

Thank you for your valuable comment. We have revised this section. Please refer page 7, lines 167-172.

3- Please structure the Methods section with appropriate subheadings for clarity.

Thank you for your valuable comment. We have structured the method and material section. Please refer page 6, lines 134, 137, and 151.

***Results***

1- Do you mean by RMANOVA, "Repeated Measures Analysis of Variance"? Please clarify.

Thank you for your comment. Yes, Repeated Measures Analysis of Variance (RMANOVA). We have added it to the result section. Please refer page 9, line 218.

2- The limited sample size of intervention phase (n = 16) raises concerns about the statistical power and generalizability of the study's findings!!! In the discussion section, please address how the small sample size may have influenced your findings and clarify how this limitation should inform the interpretation of your results.

Thank you for your insightful comments regarding the limited sample size in the intervention phase. please refer to page 19, lines 471-481.

3-In qualitative research, sample size is typically determined by the principle of theoretical saturation. However, in this study, the inclusion of 16 participants appears to have been driven primarily by the number of individuals involved in the intervention phase, rather than by saturation criteria.

Thank you for your comment. We have revised this section in the manuscript. please refer to page 6, lines 152-156.

4-The alignment between the interview questions and the reported findings requires further clarification. Specifically, the questioning guide includes prompts about both positive and negative aspects of the program (e.g., perceived barriers, potential negative contributions from newly joined participants). However, the extracted themes presented in the results section appear to focus exclusively on positive outcomes. This raises concerns about whether the analysis fully captured the range of participant responses or if negative perspectives were underrepresented or omitted. Clarify whether any negative or critical responses were provided by participants and, if so, explain how these were handled during the thematic analysis. If no negative themes emerged, consider discussing this in the limitations or discussion section, including possible reasons (e.g., social desirability bias, group dynamics). Ensure that the themes accurately reflect the scope of the questions asked and the diversity of participant perspectives.

Thank you for highlighting the need to clarify the alignment between our interview questions and reported findings. While our interview guide specifically included prompts addressing both positive and negative aspects of the HoSIP program—such as perceived barriers and potential negative influences from newly joined participants—none of the participants expressed significant negative points regarding the long-term effects or sustainability of HoSIP.

We acknowledge that the absence of negative themes may be influenced by factors such as social desirability bias, group dynamics, or the voluntary nature of participation, which may have led participants to emphasize positive experiences. We have now addressed this issue in the Discussion section as a limitation, and we confirm that our coding and analysis process systematically considered all participant responses, including any critical or negative feedback. please refer to page 19, lines 485-490.

5-The final section of the results includes two parts—'Engagement and Expansion' and 'Challenges and Adjustments.' It is unclear whether these are intended as results of the study, and if so, whether they are based on quantitative or qualitative data. Since these sections primarily describe the intervention process and group conditions, their placement within the results may be confusing to readers. It may be more appropriate to relocate this content to the methodology or discussion sections, where it can be contextualized more effectively.

Thank you for your comment. These parts were not related to quantitative or qualitative results. It just based on the researcher ‘s observation. We have embedded this section in the discussion section. Please refer to page 18, lines 440-442; lines 443-454; lines 461-470.

***Discussion***

1- While the effectiveness of interventions in reducing social isolation among older adults has been well-documented, the distinctive strength of the present study lies in the sustained impact of the intervention observed over a two-year period. This long-term effectiveness is a significant contribution and should be emphasized consistently throughout the manuscript, particularly in the discussion section.

Thank you for your comments. We have revised the discussion section to highlight the long-term effectiveness and sustainability observed over the two-year follow-up period. Please refer to page 17, lines 480-412; lines 425-426.

2- The discussion section would benefit from a more thorough and transparent acknowledgment of the study’s methodological limitations. Currently, the limitations are either briefly mentioned or not addressed in sufficient depth. Given the nature of the study, several methodological constraints—such as small sample size, lack of control group, potential biases, limited generalizability—should be explicitly discussed.

Thank you for your comments. We have revised the limitation section. please refer to page 19, lines 471-490.
